# Deciphering the genetic diversity in the Arabian Peninsula and Africa: insights from Y-STR data

**DOI:** 10.1007/s12024-025-01115-3

**Published:** 2025-11-06

**Authors:** Abdullah Hadi, Shams Hadi, Eida Khalaf Almohammed, Hayder Lazim

**Affiliations:** 1 Royal Preston Hospital, Sharoe Green Lane, Fulwood, Preston, PR2 9HT UK; 2 School of Medicine, University of Lancashire, 135A Adelphi St, Preston, PR1 7BH UK; 3 Ministry of Interior of Qatar, Doha, Qatar, Department of Biomedical Sciences, College of Health Sciences, QU Health, Qatar University, Doha, Qatar; 4 Faculty of Health, Social Care and Medicine (FHSCM), School of Medicine, Edge Hill University, Ormskirk, L39 4QP UK

**Keywords:** Y-Chromosome; Middle East; African gene flow; Y-Haplogroups; Jewish population; Ancestry variability

## Abstract

Middle Eastern and African populations make up a significant portion of the global population and exhibit substantial genetic diversity. However, genetic studies on these populations have been largely underrepresented compared to other populations. This study analysed published Y-STR data for 186 populations and regions, including 14,504 individuals from 52 Middle Eastern and 134 African populations. The highest genetic diversity was found at the DYS458 locus in the Middle East and North Africa, and at the DYS385b locus in other African regions. Multidimensional scaling (MDS) analysis and genetic distance calculations between Middle Eastern and African populations revealed five distinct clusters. The Arabian Peninsula countries formed two small clusters, while most African countries formed two mains centrally located clusters. The most common haplogroups in the Middle Eastern populations were J1a (29.4%), while in the African populations, E1b1a (43.2%) was the most prevalent. This study examined two allelic richness parameters: distinct and private alleles. Central Africa showed the highest levels of distinct alleles, with the Middle East having the third-highest level. The prevalence of private alleles in the Middle East was moderate, lower than South Africa but higher than North Africa. A population Q-matrix graph was constructed, yielding 10 clusters (K = 10) that identified population clusters in the Y-STR data corresponding to specific geographical regions and revealed stronger sub-grouping of countries within each population.

**Supplementary Information:**

The online version contains supplementary material available at 10.1007/s12024-025-01115-3.

## Introduction

Y-chromosome analysis is crucial in population genetics due to its non-recombining, unipaternally inherited nature [1]. Y chromosome Short Tandem Repeat (Y-STR) markers are particularly valuable in forensic cases, especially sexual assaults with mixed DNA samples containing minimal male DNA alongside abundant female DNA [2, 3]. They also aid in studying paternal evolutionary history [4] and molecular anthropology [5]. Since males are hemizygous for Y-chromosome markers, profiles typically show single alleles per locus. Multiple alleles indicate contributions from multiple males. Beyond establishing paternal relationships, Y-STR analysis provides geographical ancestry information for male DNA contributors, proving essential in missing person investigations [6].

Genetic studies focusing on the Y chromosome Short Tandem Repeat (Y-STR) analysis have provided valuable insights into the population history, genetic diversity, and migration patterns of populations in Africa and the Middle East. These studies have utilized various analytical approaches such as admixture analysis, phylogenetic analysis, network analysis, and haplogroup analysis to unravel the complex genetic landscape of these regions. By examining the Y chromosome researchers have been able to trace the paternal lineage of populations and better understand the genetic relationships between different groups. Africa is widely recognized as the birthplace of Homo sapiens. This conclusion is supported by anthropological and DNA evidence, indicating that populations worldwide can be traced back to an African origin approximately 200,000 years ago [7–10].

According to the Out-of-Africa model, anatomically modern humans initially expanded into the Arabian Peninsula and the Levant during the terminal Middle Pleistocene, with ongoing interaction with Africans until the Late Pleistocene. This led to two waves of modern human dispersal into Eurasia, offering a potential resolution for current archaeological, genetic, and paleontological evidence [11].

The Middle East, situated at the crossroads of Africa, Europe, and South Asia, has garnered recognition for harbouring some of the most ancient evidence of modern human presence outside of Africa, notably in the regions of the Levant and Northwest Arabia, with established dates of at least 177 thousand years ago (kya) and approximately 85 kya. Consequently, the Middle East assumes a pivotal role in elucidating the intricacies of human evolution, historical developments, and migratory patterns [12, 13].

In this study, we present a comprehensive database comprising 17 Y-STR loci from Middle Eastern and African populations to examine genetic and phylogenetic relationships between different African regions and the Middle East. We analyzed ancestry variability, allelic richness, and informativeness of genetic markers across populations. Additionally, we investigated migration patterns and routes within Africa using Y-STR data to provide insights into the demographic history and population structure of these regions. This analysis contributes to the understanding of paternal lineage diversity and evolutionary relationships in populations that remain underrepresented in genomic databases.

## Material and methods

Published Y-STR data from 186 populations and regions, comprising 14,504 individuals, were used in this study: 52 Middle Eastern populations (5,568 individuals) [14] and 134 African populations (8,936 individuals) [15–47]. These populations represent 13 Middle Eastern countries and 30 African countries.

Three main kits were used to generate the published data: the AmpFLSTR™ Yfiler™ PCR Amplification Kit, the PowerPlex® Y23 System, and the Yfiler™ Plus PCR Amplification Kit. These kits analyze 17, 23, and 27 Y-STR markers, respectively. For this study, we used the set of 17 markers from the AmpFLSTR™ Yfiler™ PCR Amplification Kit that are common to all three kits. This approach allowed us to include the largest possible number of populations in our analysis.

### Statistical analyses

#### Population genetic and phylogenetic analysis

The population genetic structure within our dataset was assessed using the analysis of molecular variance (AMOVA) approach. Calculations were performed with the Arlequin v3.5.2.2 software [48, 49], which enabled us to determine the average pairwise differences both between populations (PiXY) and within populations (PiX). We also calculated the corrected average pairwise difference between populations as PiXY − (PiX + PiY)/2.

To further quantify genetic distances between populations, we calculated pairwise genetic distances (R_ST_) based on the Y-STR data. To investigate genetic similarities and visualize the variance in genetic differences among populations, we conducted a multidimensional scaling (MDS) analysis. For phylogenetic tree construction, we used allele frequency data with the POPTREE2 online tool [50] in conjunction with the FigTree software [51].

#### Allelic richness in different population groups

To gain a more comprehensive understanding of genetic diversity and population relationships, this study investigated allele distributions across six distinct regions: the Middle East, North Africa, East Africa, West Africa, South Africa, and African populations residing outside of Africa. The analysis focused on assessing both the number of distinct alleles present within each population and the number of alleles exclusive to a particular population (referred to as private alleles), which are not observed in any other populations. These two fundamental characteristics serve as valuable indicators when examining populations at a specific locus, especially when analyzing highly variable multiallelic markers such as microsatellites. The Allelic Diversity Analyzer (ADZE)v1.0 software was used to accurately quantify the counts of distinct and private alleles [52, 53].

#### Informativeness statistics for genetic markers

This study also used the program Infocalc, which calculates informativeness statistics for genetic markers used in ancestry inference. Three parameters were assessed: first, the informativeness for assignment (In), which measures the amount of information gained about population assignment from observing a single, randomly chosen allele at a locus; second, the informativeness for ancestry coefficients (Ia), which quantifies the information gained about ancestry coefficients from such an observation; and finally, the optimal rate of correct assignment (ORCA), which represents the highest possible rate at which a randomly chosen allele from a pooled collection of populations can be correctly assigned to its most likely source population [54–56].

#### Haplogroups and median-joining networks

The haplogroups of the study populations were determined using NevGen Genealogy Tools v1.1. [57]. Additionally, the NevGen Probability Calculator for Time to Most Recent Common Ancestor (TMRCA) was used to estimate the TMRCA for the J1a haplogroup in Arabia and to identify the most frequent value as the ancestral haplotype. The TMRCA calculations in NevGen take into account backward Y-STR mutations, and also multiple-step mutations.

Median-joining networks were constructed for the most common haplogroups using Network 10.2.0.0 and Network Publisher [58, 59]. In cases involving intermediate alleles, repeat numbers were rounded to the nearest integer. Additionally, for the purpose of network construction, constitutively duplicated loci (DYS385a,b) were excluded from the analysis.

#### *Population structure*

This study examined the population structure of 186 Middle Eastern and African populations, encompassing a total of 14,504 individuals. The analysis was conducted using STRUCTURE v2.3.7 with an admixture model [60, 61].

To process the output and evaluate probability values across a wide range of K values, the STRUCTURE HARVESTER program was used. This program also helped identify the optimal number of genetic clusters that best fit the data [62, 63]. To consolidate the findings, multiple iterative analyses were performed on each dataset, and the results were aligned using CLUMPP [64, 65]. These aligned results were then used to generate the population Q-matrix graph with Distruct [64, 66].

#### Ancestry variability analysis

The ancestry variability in this study examined the variation in membership coefficients among individuals assigned to the designated clusters. The FSTruct program was used to investigate differences in membership coefficient variability between admixed and non-admixed populations [67, 68].

#### *Migration and gene flow in Africa*

The Migrate program was used to examine gene flow between different geographical regions of North, East, Central, West, and South Africa [69]. Three models were applied: Model 1 involved unidirectional gene flow from one population to another; Model 2 accounted for divergence from a common ancestral population; and Model 3 incorporated both divergence from the ancestral population and ongoing immigration. Gene flow calculations were performed using pairs of regions, with all three models applied in both directions. This approach enabled comprehensive coverage of the entire African continent.

## Results

### Population genetic and phylogenetic analysis

**Fig. 1:**
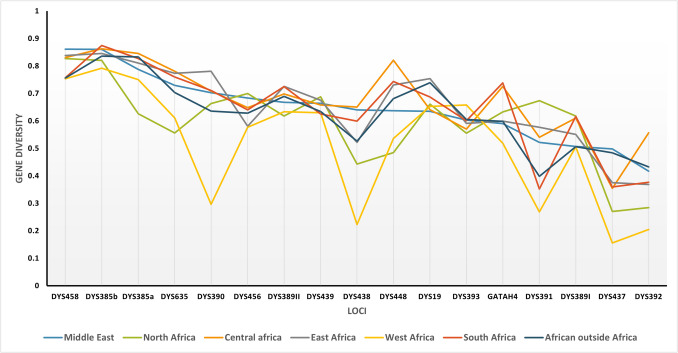
The genetic diversities of the 17 Y-STR loci in the Middle Eastern populations, the five African geographical regions (North, West, East, Central and South) and Africans outside Africa

Genetic diversity for the 17 Y-STR loci was calculated among Middle Eastern populations, the five African geographical regions (North, West, East, Central, and South Africa), and African populations residing outside Africa. The locus DYS458 exhibited the highest genetic diversity in Middle Eastern and North African populations. In all other African populations, the locus DYS385b showed the highest genetic diversity. The lowest genetic diversity in the Middle East and East Africa was observed at locus DYS392, while DYS437 showed the lowest genetic diversity in North, Central, and West Africa. In both South Africa and African populations outside Africa, the locus DYS391 exhibited the lowest genetic diversity. The results are presented in Fig. 1 and Supplementary Table S1.

**Fig. 2:**
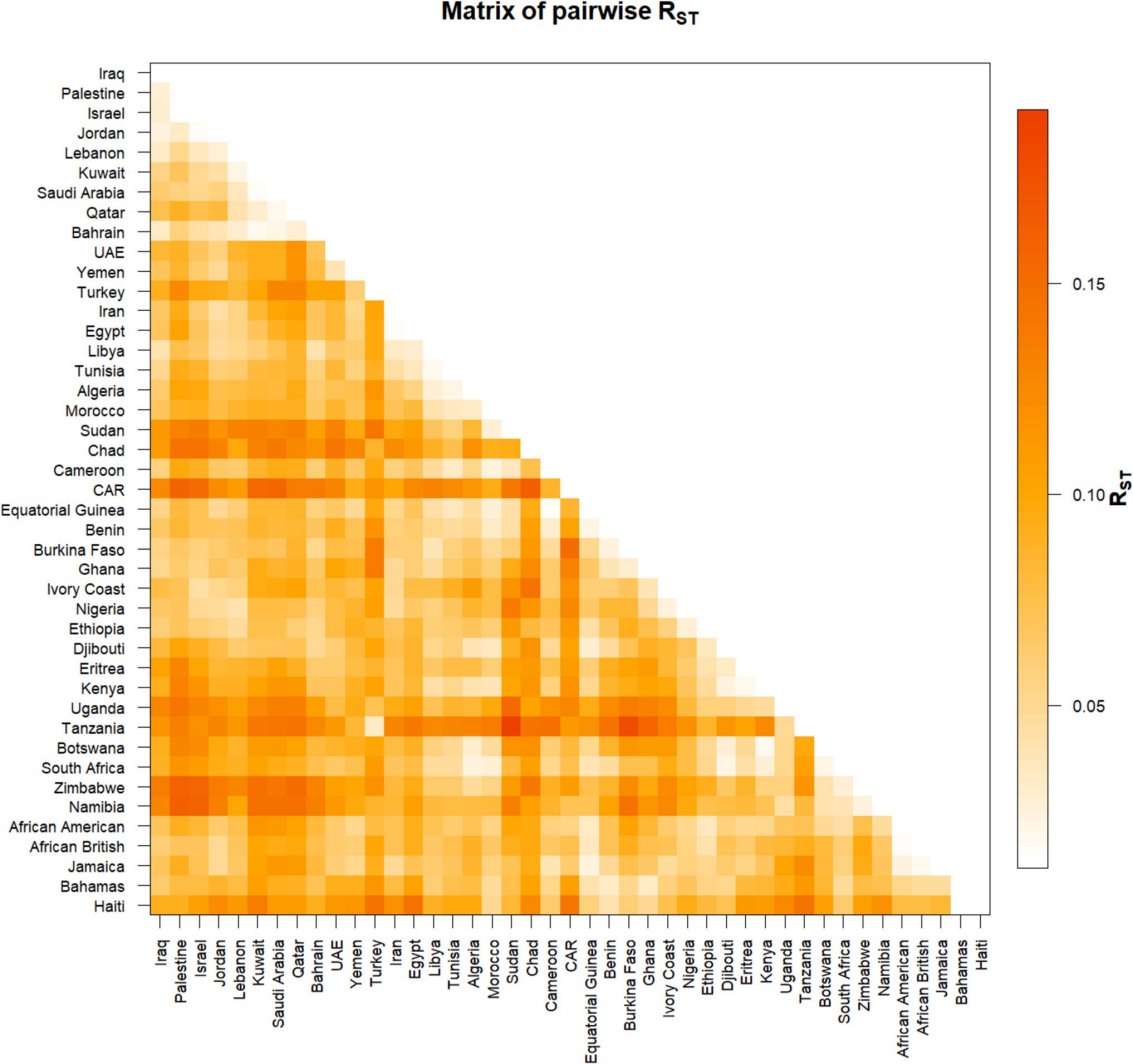
The matrix of pairwise genetic distance R_ST_ of Y-STR between the Middle Eastern and African populations based on 17 Y-STR markers. This matrix was generated using Arlequin v3.5.2.2 software. CAR: Central African Republic, UAE: United Arab Emirates

A pairwise matrix plot of R_ST_ distances was generated to compare 43 populations—13 from the Middle East and 30 from Africa. Among Middle Eastern populations, the closest pair was Israel and Palestine (R_ST_ = 0.01365), while the most distant were Qatar and Turkey (R_ST_ = 0.12883). In Africa, the closest populations were from the Bahamas and Haiti, with an R_ST_ of 0.01138; this pair was also the closest overall across both Africa and the Middle East. Conversely, the most distant populations were Tanzania and Sudan, with an R_ST_ of 0.19113, making them the most genetically distant pair across both regions. The results are presented in Supplementary Table S2 and Fig. 2.

The average pairwise differences were examined to estimate the corrected genetic differences among 43 populations. These differences were specifically analysed in three contexts: between the populations as a whole, within each individual population, and between different populations using Nei’s distance.

For the average number of pairwise differences between populations, the lowest value was observed between Jordan and Israel (164.61448), while the highest was recorded between the Central African Republic and Turkey (272.2404). Within the Middle East, the lowest value was again between Jordan and Israel, and the highest was between Turkey and the UAE (238.23841). In Africa, the lowest value was found between Kenya and Djibouti (168.01477), while the highest was between the Central African Republic and Chad (271.70886).

For within-population calculations across both the Middle East and Africa, Djibouti had the lowest value (160.13153), while the Central African Republic had the highest (241.80979). In the Middle East specifically, Jordan had the lowest value (161.72676), and Turkey had the highest (239.42689).

**Fig. 3:**
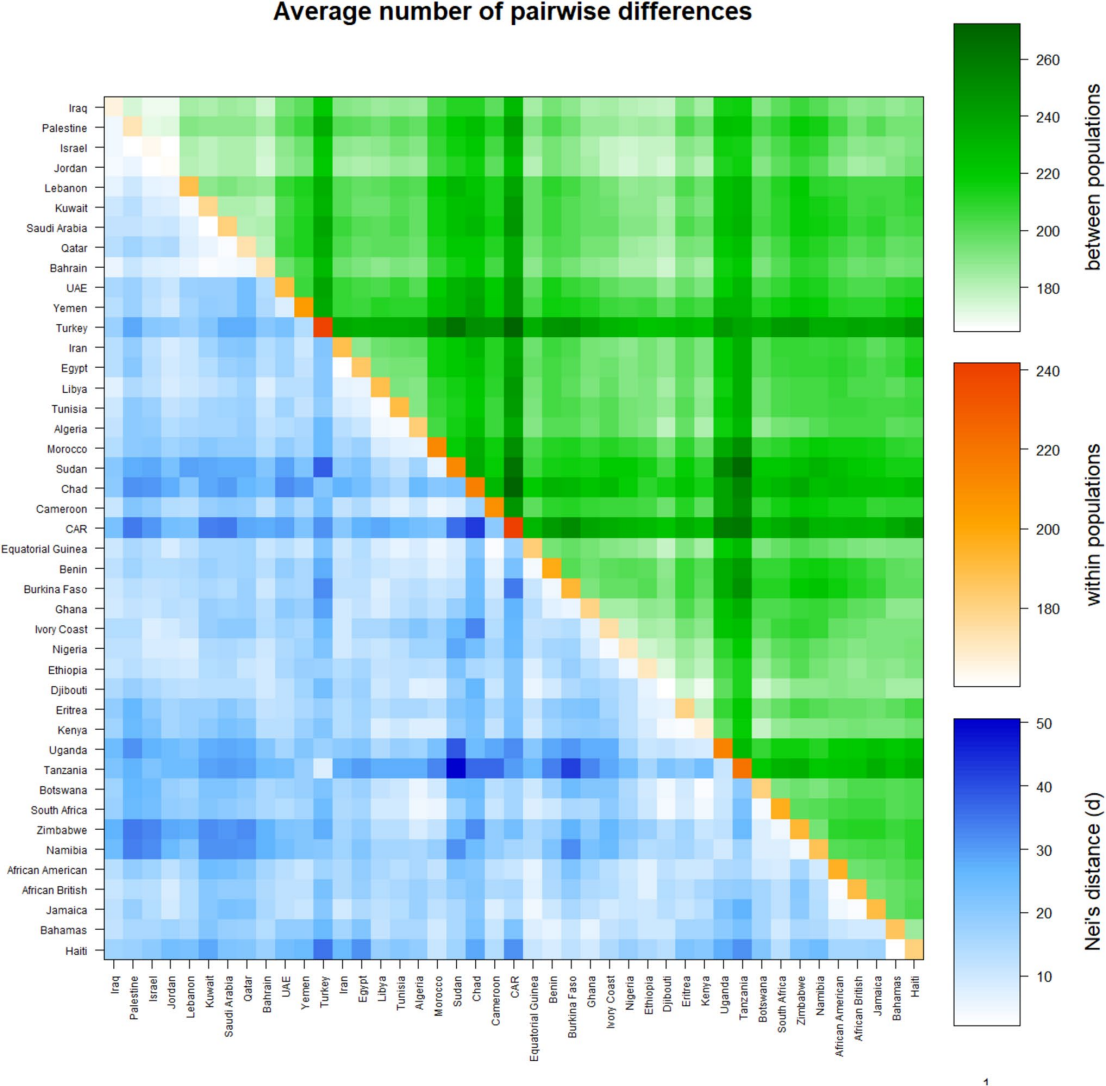
Matrix plot showing population average pairwise differences based on 17 loci. The area above the diagonal (green) shows the average number of pairwise differences between populations; the diagonal (orange) shows the average number of pairwise differences within population; and below the diagonal (blue) shows the corrected average pairwise difference. The scale of differences is shown on the right side of the matrix

Regarding Nei’s distance, for both the entire dataset and the African populations, the lowest value was found between the Bahamas and Haiti (2.13661), while the highest was observed between Sudan and Tanzania (50.67652). In the Middle East, Palestine had both the lowest and highest Nei’s distance values: the lowest with Israel (2.3199) and the highest with Turkey (28.22702). The results are shown in Supplementary Table S3 and Fig. 3.

**Fig. 4:**
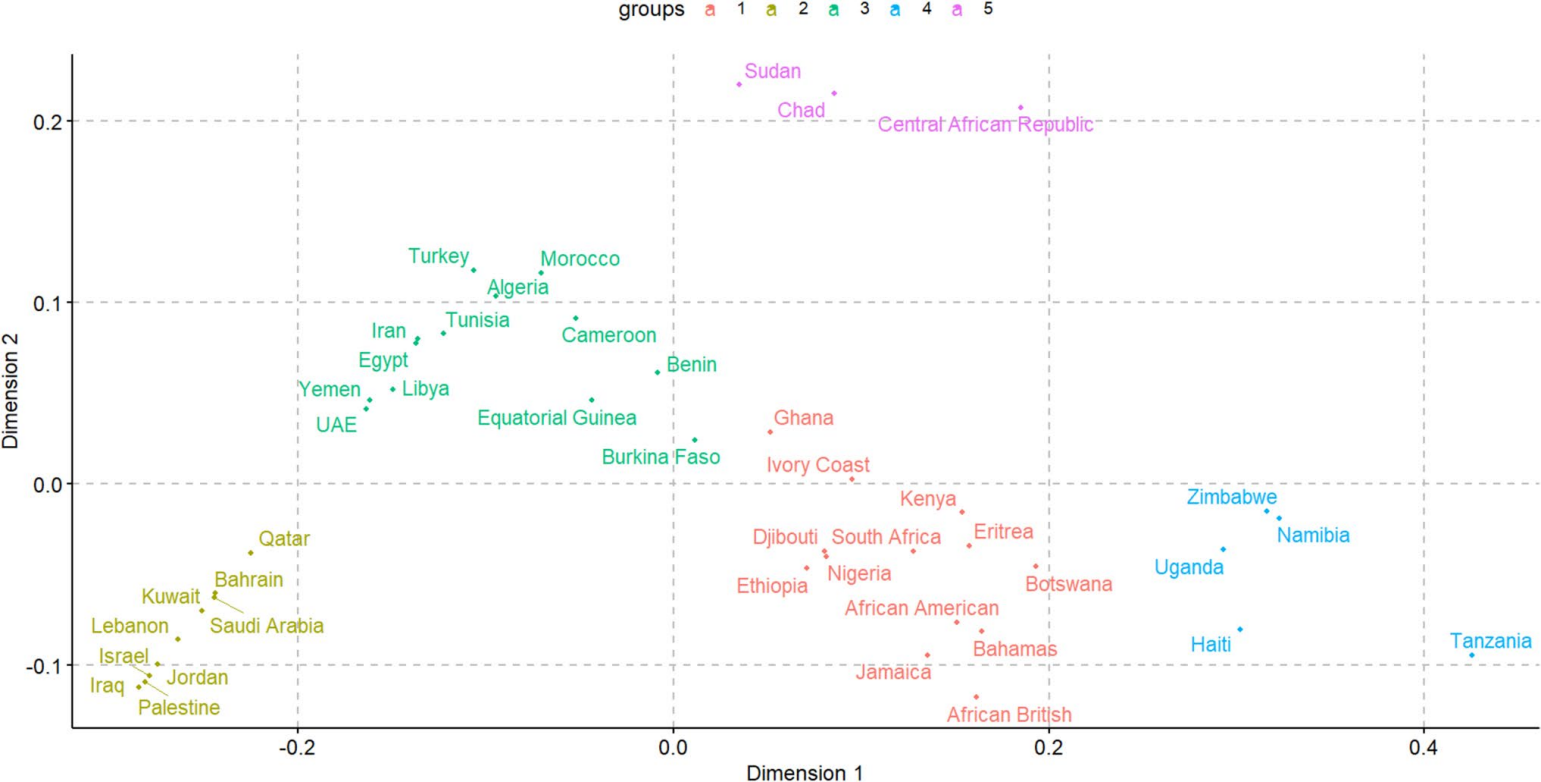
Multidimensional scaling (MDS) plots comparing the Middle Eastern and the African populations based on 17 Y-STR markers. Five clusters could be identified

Multidimensional scaling (MDS) and genetic distance analyses were performed among all Middle Eastern and African populations. Five clusters were identified and distributed as follows: countries of the Arabian Peninsula were located in the lower left quadrant. Most African countries formed two main, centrally located clusters. The first cluster, composed of North African countries, was situated in the central upper left quadrant; this cluster also included four Middle Eastern countries—Yemen, UAE, Iran, and Turkey. The second cluster appeared in the central lower right quadrant, where African populations outside Africa merged with most other African countries. The remaining African countries formed a smaller fourth cluster in the lower right quadrant. The fifth cluster, located in the upper right quadrant, consisted of three African countries: Sudan, Chad, and the Central African Republic. The MDS results are shown in Fig. 4.

A three-dimensional MDS was generated to get a better idea about these five clusters; it could be found as an interactive open-source which can be viewed in Supplementary Figure S1.

**Fig. 5:**
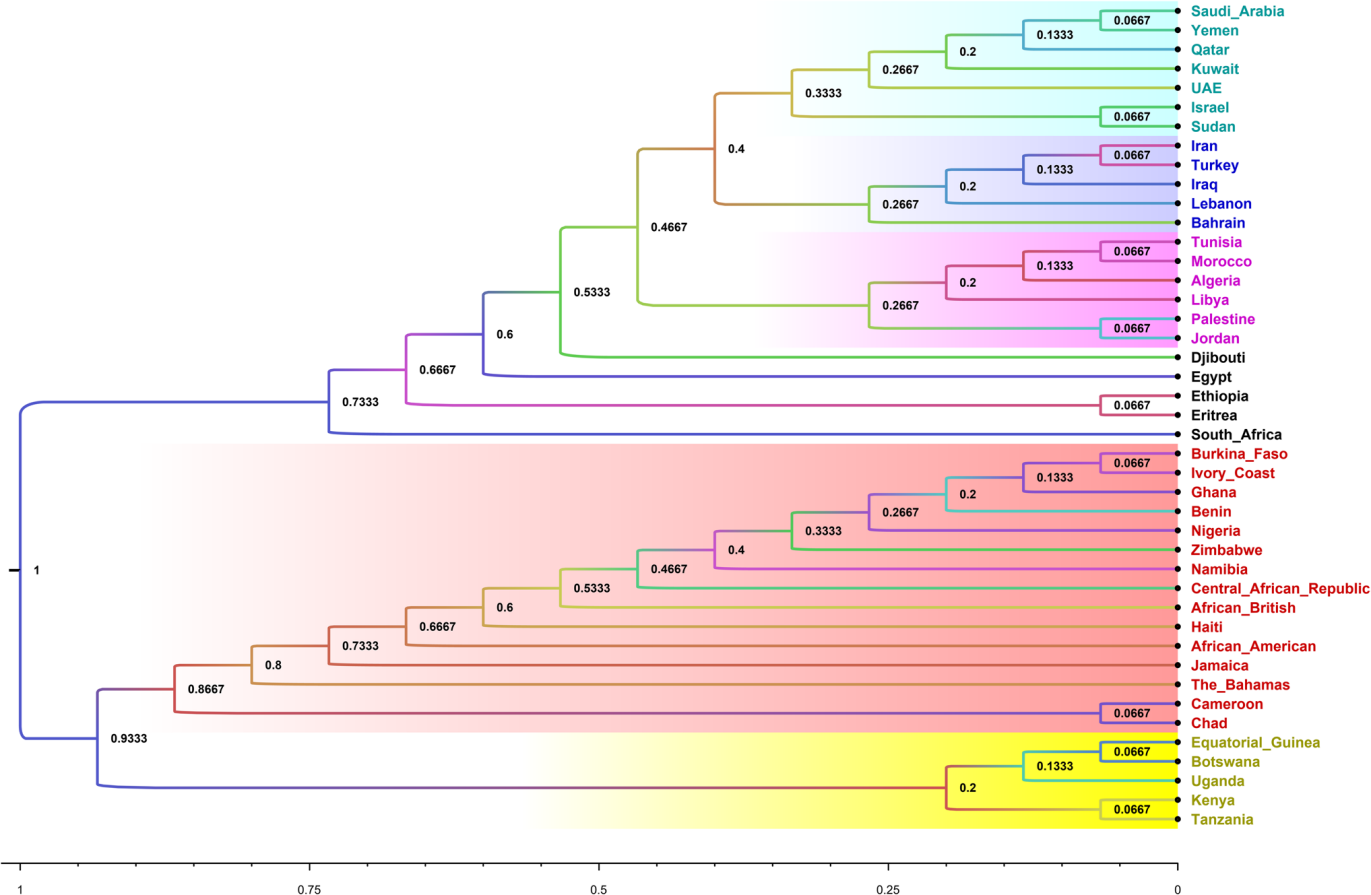
Phylogenetic tree of genetic relationships among the 43 Middle Eastern and African populations. Five clusters (K = 5) were created. The red-coloured cluster is the largest (15 populations), followed by the cyan-coloured cluster (7 populations), and the purple-coloured cluster (6 populations). The remaining two clusters each have five populations. This phylogenetic tree was generated using POPTREE2 software

To assess the diversity between African and Middle Eastern populations, a phylogenetic tree was constructed. Five subpopulations (K = 5) represented the optimal clustering for the 43 populations analyzed (Fig. 5). The Middle Eastern populations formed two small clusters, with Sudan—an African country—joining one of these clusters. The North African countries formed a third cluster, which also included Palestine and Jordan. The fourth and fifth clusters encompassed the majority of the African countries, with the fourth being the largest cluster.

### Allelic richness in Middle East and Africa

**Fig. 6:**
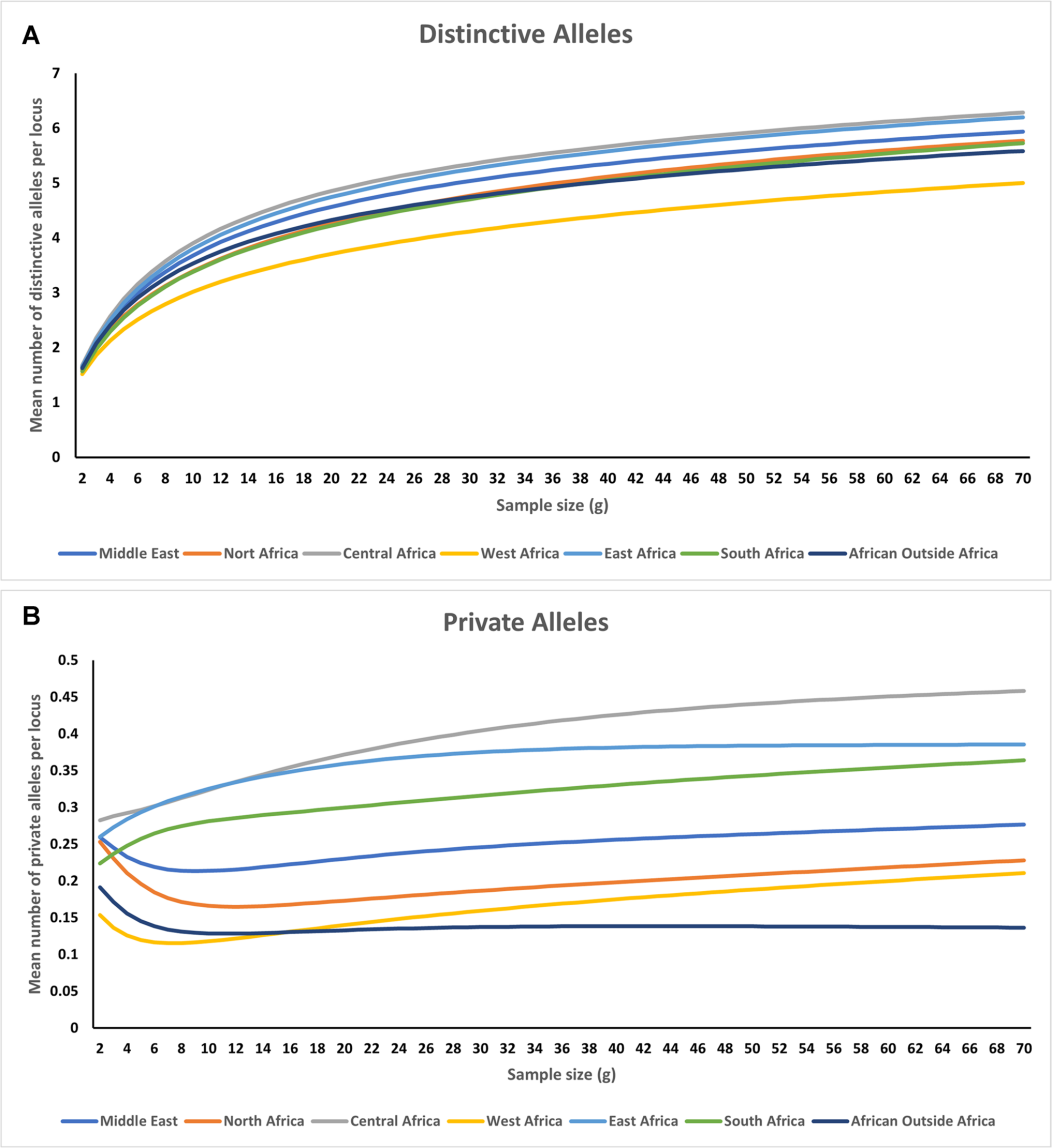
The mean number of (**A**) distinct alleles per locus and (**B**) private alleles per locus of the seven regions: Middle East, North Africa, Central Africa, West Africa, East Africa, South Africa and Africans outside Africa

This study examined two measures of allelic richness, distinct alleles and private alleles, across the Middle East and five regions of Africa (North, Central, East, West, and South). The findings showed that Central Africa had the highest level of distinct alleles, while West Africa had the lowest. The Middle East ranked third in the number of distinct alleles (Fig. 6A and Supplementary Table S4).

Additionally, the analysis revealed that Central Africa exhibited the highest levels of private alleles, whereas populations of African descent living outside Africa had the lowest levels. The Middle East was intermediate, ranking below South Africa but above North Africa (Fig. 6B and Supplementary Table S5).

### Informativeness statistics for genetic markers

To assess the informativeness (In) of genetic markers for distinguishing between Africa and the Middle East, two markers, DYS438 and DYS390, stood out with high values of 0.25 and 0.24, respectively (Supplementary table S6a). Within the Middle Eastern population specifically, the marker DYS458 showed the highest informativeness with a value of 0.34, followed by DYS456 with a value of 0.14 (Supplementary table S6b).

### Haplogroups and median-joining networks

The most common haplogroups among Middle Eastern populations are J1a (29.4%, 1637/5568 individuals), J2a (17.9%, 999/5568), E1b1b (11%, 613/5568), R1a (9.8%, 551/5568), and G2a (4.2%, 239/5568). Median-joining networks were constructed for each of these haplogroups to show their distribution among the Middle Eastern populations (Supplementary Figure S2).

The most common haplogroups among African populations are E1b1a (42.2%, 3,774/8936 individuals), E1b1b (21.6%, 1935/8936), and R1b (8.5%, 761/8936).

NevGen probability calculator of Time to Most Recent Ancestor (TMRCA) was used to calculate the generations and most frequent value as ancestor haplotype of main Middle Eastern haplogroup J1a. Iraq was the highest with 167 generations followed by Yemen and Palestine 166 and 163 generations respectively. The lowest generation number was noticed in Saudi Arabia and Israel 96 and 95 generations respectively. The differences between the ancestor haplotypes was noticed to involve nine markers: DYS456, DYS390, DYS389II, DYS458, DYS385a, DYS385b, DYS391, DYS439 and DYS635. Table 1 shows the calculations of TMRCA for the haplogroup J1a of the countries in Arabia.

**Table 1:** The calculations of TMRCA, the generations and most frequent value as ancestral haplotype of the countries in Arabia for the haplogroup J1a. The order of the loci in the most frequent values for the haplotypes are: DYS456, DYS389I, DYS390, DYS389II, DYS458, DYS19, DYS385a, DYS385b, DYS393, DYS391, DYS439, DYS635, DYS392, GATAH4, DYS437, DYS438, DYS448. The differences between the ancestral haplotypes were highlighted in yellow

Country	Generations	Probability %	Sum Probability %	Most frequent value as ancestral haplotype
Iraq	167	7.16715	56.2814	14 13 23 30 18 14 13 18 12 11 11 21 11 11 14 10 20
Yemen	166	4.25957	51.7964	14 13 23 30 18 14 **14** 18 12 **10** 11 21 11 11 14 10 20
Palestine	163	3.53043	53.5234	**13** 13 23 **29 20** 14 13 18 12 11 11 21 11 11 14 10 20
Jordan	163	4.86133	50.583	14 13 **22 29 19** 14 13 18 12 **10** 11 21 11 11 14 10 20
Lebanon	160	5.44218	52.6427	**15** 13 23 30 18 14 13 18 12 **10** 11 21 11 11 14 10 20
Bahrain	133	6.41968	56.0834	14 13 23 30 18 14 13 18 12 **10** 11 21 11 11 14 10 20
UAE	124	5.14747	54.5235	14 13 23 30 18 14 13 18 12 **10** 11 21 11 11 14 10 20
Qatar	117	8.27285	53.3473	14 13 23 **29** 18 14 13 18 12 11 11 21 11 11 14 10 20
Kuwait	102	6.79695	53.0673	14 13 23 30 18 14 13 **19** 12 11 11 21 11 11 14 10 20
Saudi Arabia	96	13.5289	52.8574	14 13 23 30 18 14 13 18 12 11 11 21 11 11 14 10 20
Israel	95	2.55379	50.3233	**16** 13 23 30 18 14 13 18 12 11 **10 20** 11 11 14 10 19

### Population structure

**Fig. 7:**
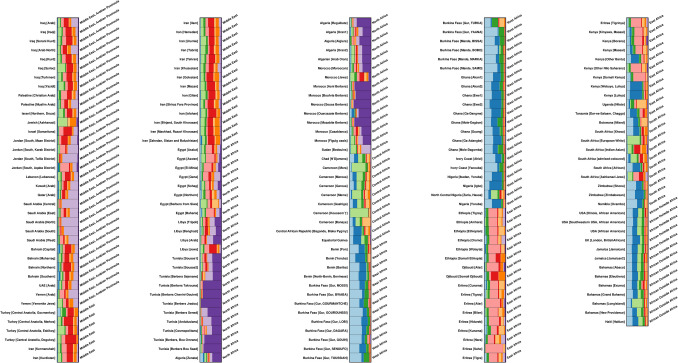
The graph of population Q- matrix of the Y-STR haplotypes using 17 STR markers from 186 Middle Eastern and African populations (14504 individuals) showing 10 clusters (K = 10)

The Y-STR graph of the populations' Q-matrix (Fig. 7 and Supplementary Table S7) revealed 10 clusters, based on 17 STR markers from 186 populations and regions (14,504 individuals), including 52 Middle Eastern and 134 African populations. The population clusters identified from the Y-STR data corresponded to specific geographical regions and showed a clear sub-grouping of countries within each cluster.

The Middle Eastern populations formed a distinct cluster. North Africa also showed a unique cluster, except for Egypt, which was more closely related to the Eastern Africa cluster. Central Africa formed its own cluster, with the exception of the Central African Republic and Equatorial Guinea, both of which overlapped with the West Africa cluster. East Africa had its own cluster as well, except for Kenya (Bantu and Luhya), which shared elements with West Africa. South Africa formed a separate cluster, except for Zimbabwe and Namibia, which showed some shared elements with West Africa. Populations of African descent living outside Africa formed their own cluster, which shared characteristics with both Central and West African countries.

### Ancestry variability analysis

**Fig. 8:**
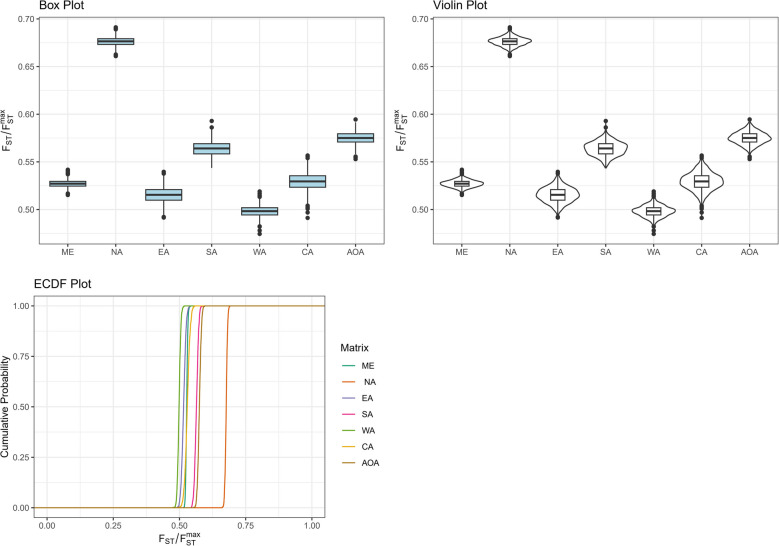
Box plot, violin plot, and empirical cumulative distribution function (ECDF) plot of the bootstrap distribution of ${\mathrm{F}}_{\mathrm{ST}}/{\mathrm{F}}_{\mathrm{ST}}^{\mathrm{max}}$ for each Q matrix in the STRUCTURE analysis for the Middle Eastern and African populations. ME: Middle East, NA: North Africa, EA: East Africa, SA: South Africa, WA: West Africa, C: Central Africa, AOA: Africans outside Africa. West and East Africa had the lowest ratio, and North Africa had the highest

The ${\mathrm{F}}_{\mathrm{ST}}/{\mathrm{F}}_{\mathrm{ST}}^{\mathrm{max}}$ ratios were used to compare the variability of Q matrices across seven regions in order to study ancestry variability among the populations. The analysis revealed that North Africa had the highest ${\mathrm{F}}_{\mathrm{ST}}/{\mathrm{F}}_{\mathrm{ST}}^{\mathrm{max}}$ ratio (0.67636), while West Africa had the lowest (0.49807). The results are presented in Fig. 8 and Supplementary Table S8.

**Fig. 9:**
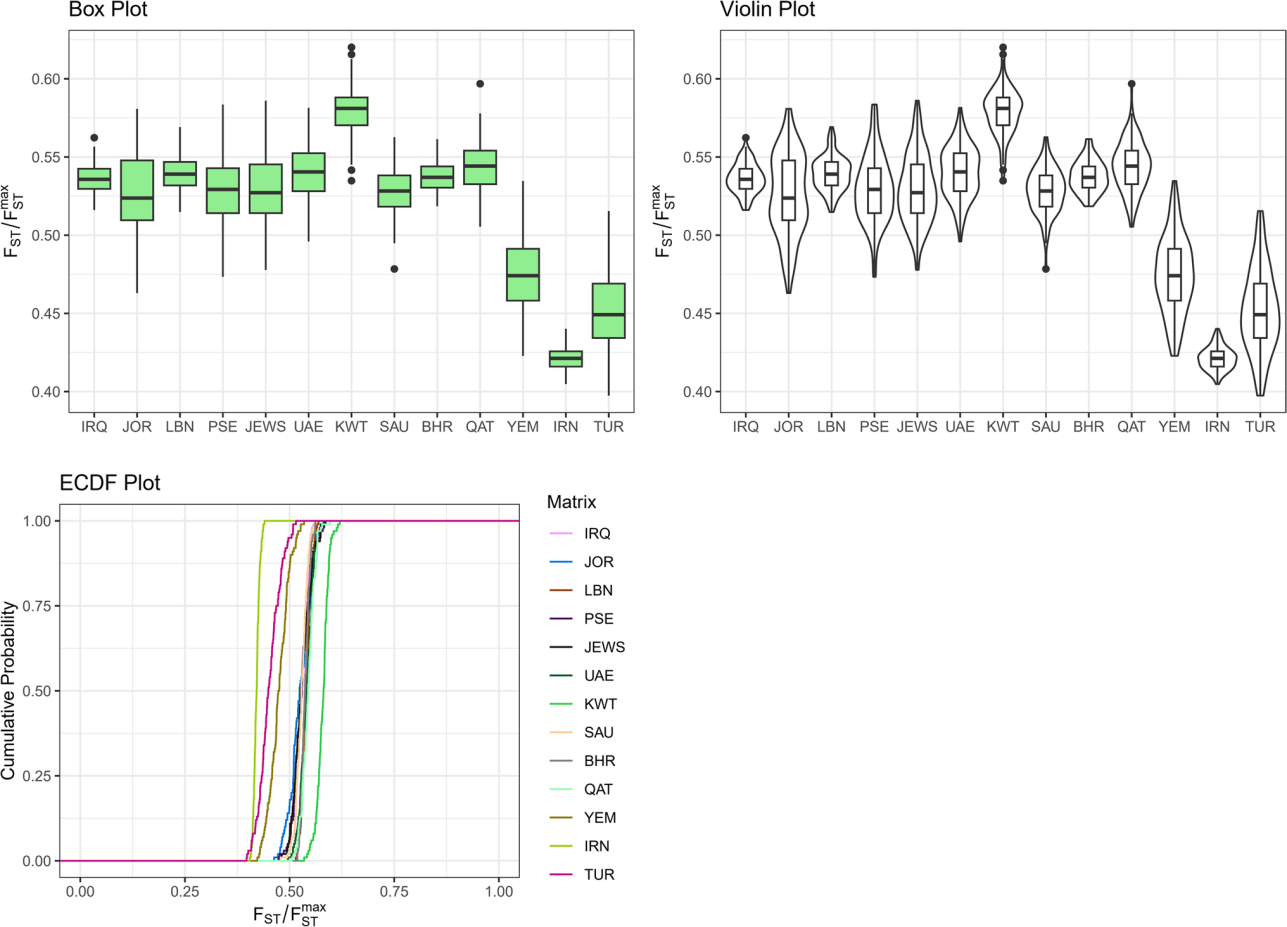
Box plot, violin plot, and empirical cumulative distribution function (ECDF) plot of the bootstrap distribution of ${\mathrm{F}}_{\mathrm{ST}}/{\mathrm{F}}_{\mathrm{ST}}^{\mathrm{max}}$ for each Q matrix in the STRUCTURE analysis for the Middle Eastern populations. IRQ: Iraq, JOR: Jordan, LBN: Lebanon, PSE: Palestine, UAE: United Arab Emirates, KWT: Kuwait, SAU: Saudi Arabia, BHR: Bahrain, QAT: Qatar, YEM: Yemen, IRN: Iran, TUR: Turkey

We analyzed ancestry variability for countries within each of the seven regions to gain a clearer understanding of regional differences. First, the Middle Eastern populations exhibited a distinct range of ${\mathrm{F}}_{\mathrm{ST}}/{\mathrm{F}}_{\mathrm{ST}}^{\mathrm{max}}$ ratios, with Kuwait showing the highest value (0.58169) and Iran the lowest (0.42201). The results also revealed that, among the Arab populations in the Middle East, the Yemeni population has the lowest ${\mathrm{F}}_{\mathrm{ST}}/{\mathrm{F}}_{\mathrm{ST}}^{\mathrm{max}}$ ratio (0.47690). The results are presented in Fig. 9 and Supplementary Table S9.

**Fig. 10:**
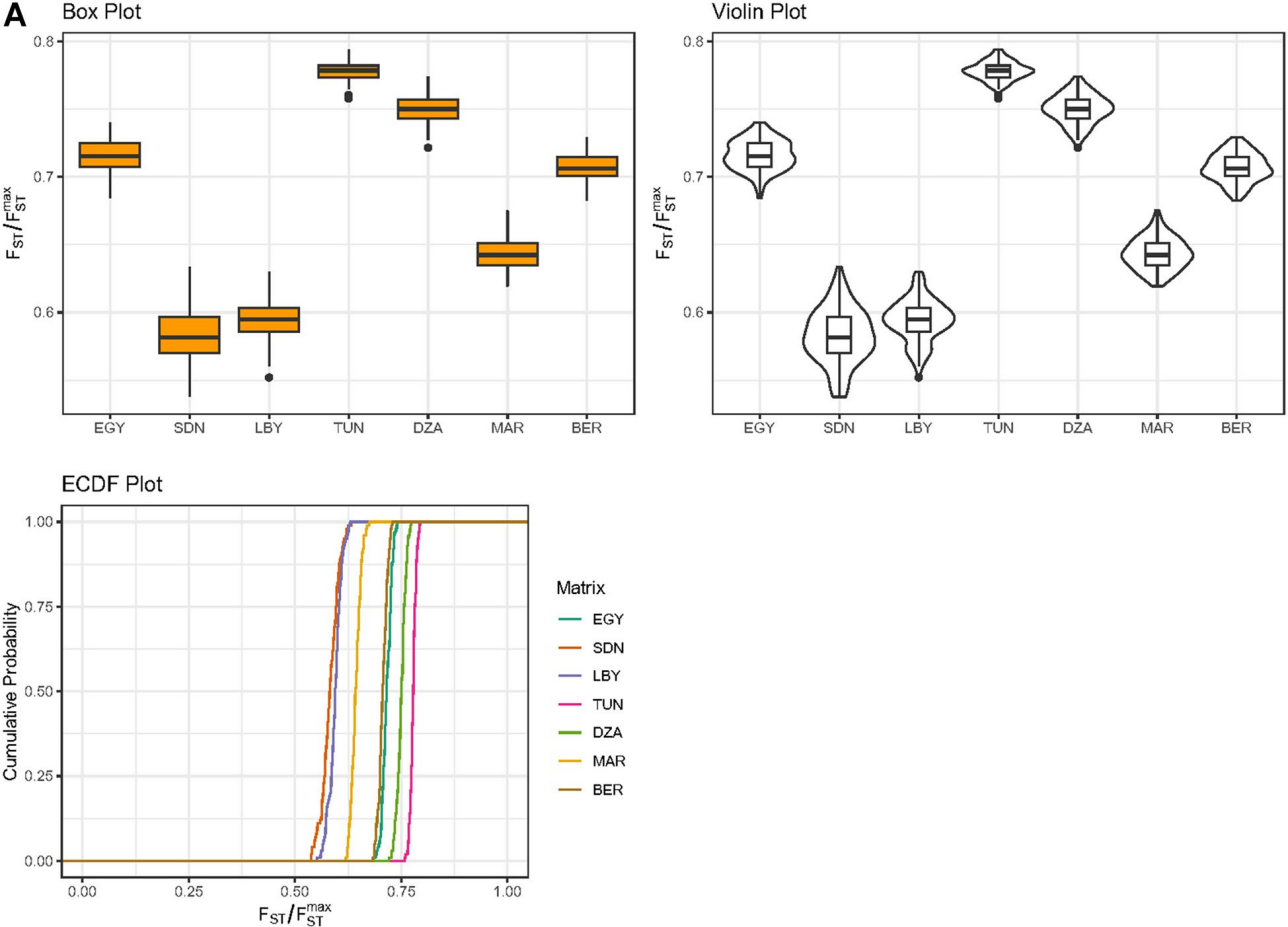

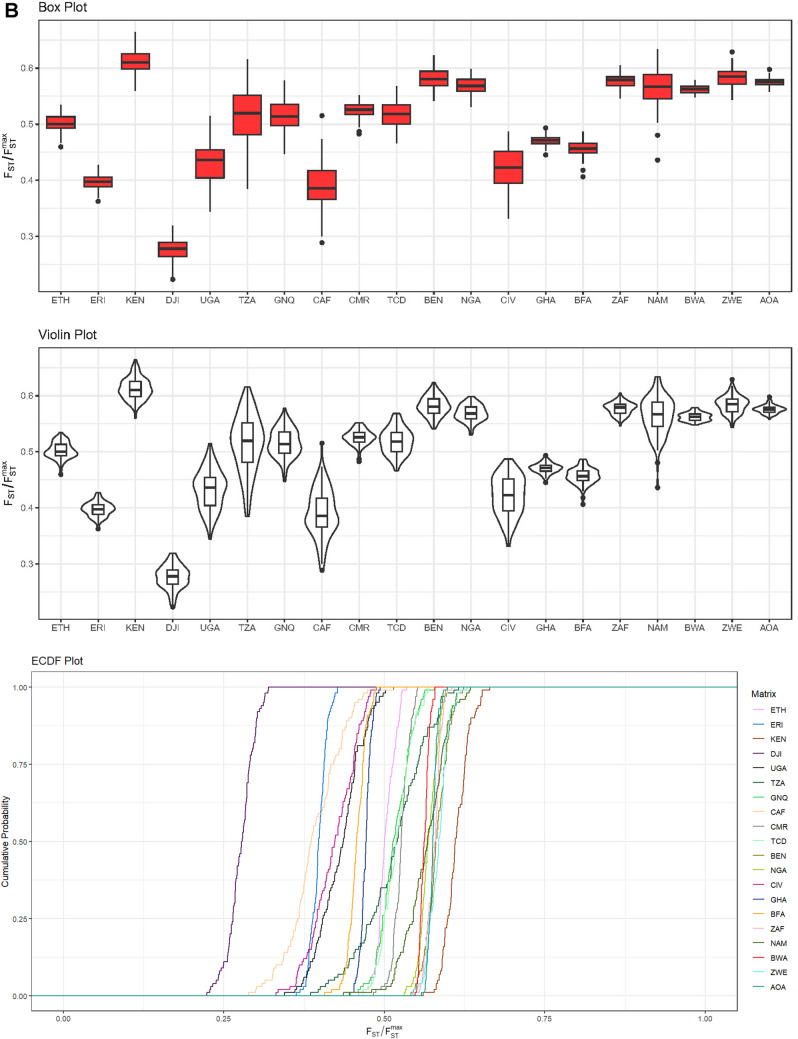
Box plot, violin plot, and empirical cumulative distribution function (ECDF) plot of the bootstrap distribution of ${\mathrm{F}}_{\mathrm{ST}}/{\mathrm{F}}_{\mathrm{ST}}^{\mathrm{max}}$ for each Q matrix in the STRUCTURE analysis for: A. North Africa: Egypt (EGY), Sudan (SDN), Libya (LBY), Tunisia (TUN), Algeria (DZA), Morocco (MAR), Berber (BER). B. Sub-Saharan Africa: East Africa [Ethiopia (ETH), Eritrea (ERI), Kenya (KEN), Djibouti (DJI), Uganda (UGA)], Central Africa [Equatorial Guinea (GNQ), Central Africa republic (CAF), Cameroon (CMR), Chad (TCD)], West Africa [Benin (BEN), Nigeria (NGA), Ivory coast (CIV), Ghana (GHA), Burkina Faso (BFA)], South Africa [Tanzania (TZA), South Africa (ZAF), Namibia (NAM), Botswana (BWA), Zimbabwe (ZWE)], Africans outside Africa (AOA)

Similarly, in North Africa, Tunisia and Sudan registered the highest and lowest ${\mathrm{F}}_{\mathrm{ST}}/{\mathrm{F}}_{\mathrm{ST}}^{\mathrm{max}}$ ratios, with values of 0.77727 and 0.58417, respectively. In the sub–Saharan Africa, the lowest ratios were found in Djibouti and Eritrea with values 0.28213 and 0.39647, respectively. While the highest ratios were found in Zimbabwe and Kenya with values 0.58631 and 0.61103, respectively. Figure 10 and supplementary tables S10 and S11 show the ${\mathrm{F}}_{\mathrm{ST}}/{\mathrm{F}}_{\mathrm{ST}}^{\mathrm{max}}$ ratios of different African countries in different regions of Africa.

**Fig. 11:**
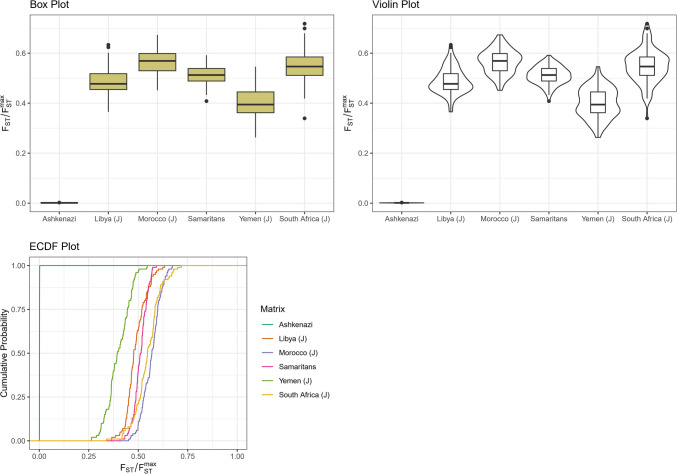
Box plot, violin plot, and empirical cumulative distribution function (ECDF) plot of the bootstrap distribution of ${\mathbf{F}}_{\mathbf{S}\mathbf{T}}/{\mathbf{F}}_{\mathbf{S}\mathbf{T}}^{\mathbf{m}\mathbf{a}\mathbf{x}}$ for each Q matrix in the STRUCTURE analysis for the Jewish populations

Finally, the six Jewish populations in this study were analysed individually, revealing that the Jews from Morocco had the highest ${\mathrm{F}}_{\mathrm{ST}}/{\mathrm{F}}_{\mathrm{ST}}^{\mathrm{max}}$ ratio (0.57191), and the Yemeni Jews had the lowest (0.41759). The ${\mathrm{F}}_{\mathrm{ST}}/{\mathrm{F}}_{\mathrm{ST}}^{\mathrm{max}}$ ratio observed in the Ashkenazi Jewish population was notably low, nearly reaching the baseline, with a recorded value of 0.00114. Figure 11 and supplementary table S12 show the ${\mathrm{F}}_{\mathrm{ST}}/{\mathrm{F}}_{\mathrm{ST}}^{\mathrm{max}}$ ratios of different Jewish populations.

### Gene flow in Africa

**Fig. 12:**
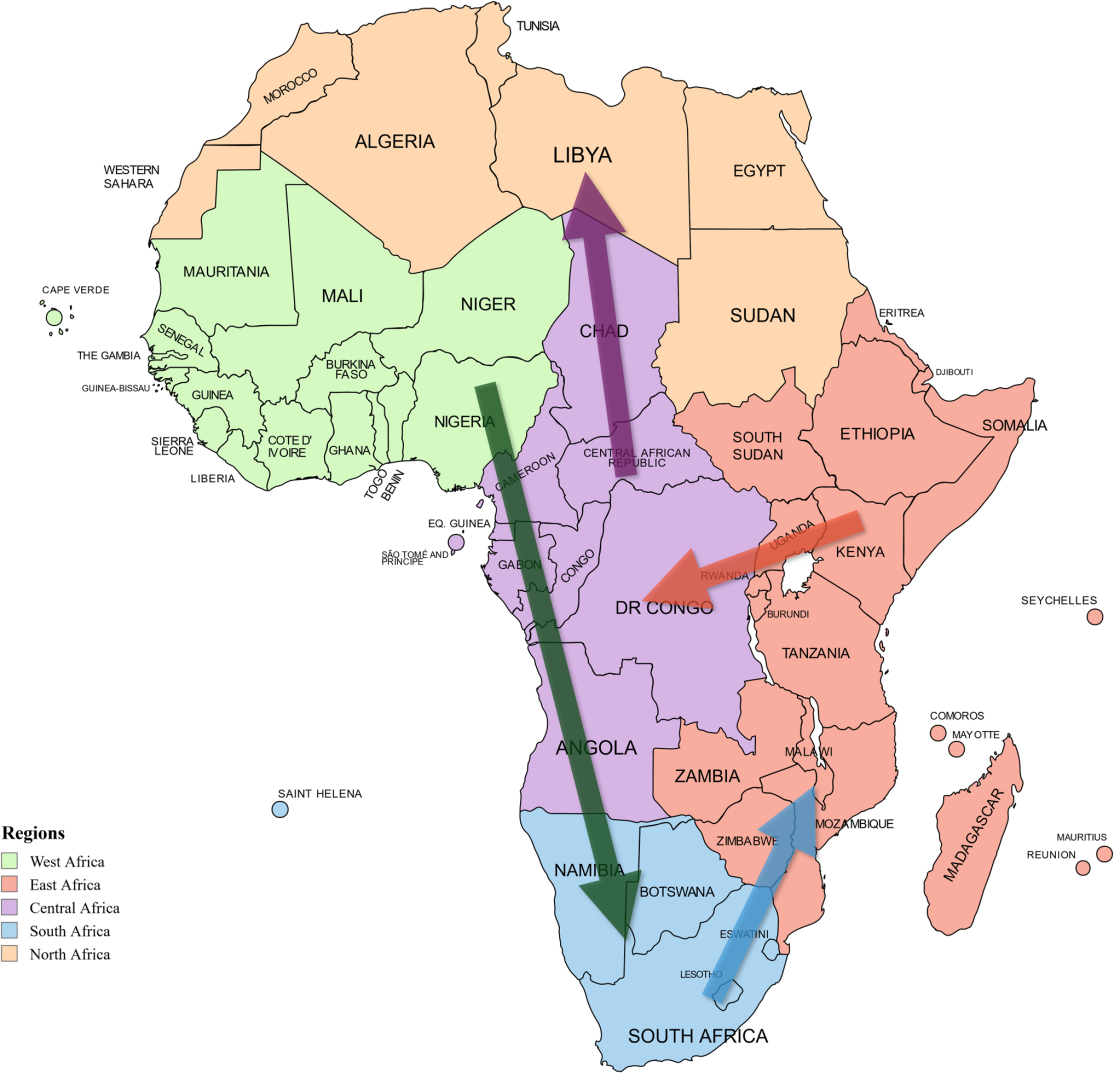
The most dominant migration routes within the African continent. The numbers in front of the dominant migration routes represent the dominant migration model. The most probable routes were: west→south1, south→ east1, east→central1 and central→north2

Across Africa, Model 1 was the most probable gene-flow route for three corridors—East to Central (0.8625), South to East (0.9385), and West to South (0.8899)—with the sole exception of the Central to North corridor, where Model 2 was overwhelmingly favoured (0.9999). The gene flow results are shown in Fig. 12 and supplementary table S13.

## Discussion

The genetic diversity calculations for the 17 Y-STR markers for the Middle Eastern and the five regions in Africa showed very distinctive patterns. In the Middle East and North Africa, the locus DYS358 showed the highest genetic diversity. In the Middle East, this is due to the presence of microvariant alleles in this locus which is also associated with the haplogroup J1 [70, 71]. The high diversity of this locus in North Africa it might be due to the impact the Arab expansion to North Africa [72].

Haplogroup J-M304 exhibits a notable prevalence in the Arabian Peninsula and Mesopotamia. This particular haplogroup undergoes a division into two subgroups: J1-M267, which has been linked to the dissemination of pastoral economies within the arid regions of West Asia, and J2-M172, which demonstrates a stronger association with agricultural practices in the northern latitudes of West Asia [73].

TMRCA calculations for the Middle Eastern population indicate that the Levant and northern Arabia are older regions compared to the southern part of Arabia, with the exception of Yemen. This finding supports the proposed migration route within Arabia, suggesting that the Levantine corridor was likely the main passageway out of Africa [71].

Studying the TMRCA in Middle Eastern population showed that 9 out of the 17 markers exhibited mutations. Six of these markers ranked in the top half for marker informativeness, and their occurrence was not related to the markers’ genetic diversity rankings.

The abundance of distinctive and private alleles in Africa supports the hypothesis that human evolution originated in Africa and subsequently spread to other parts of the world through a series of founder events. The regions of Africa and the Middle East, which are geographically connected, exhibit the highest diversity of alleles. As the migration of humans from Africa occurred in stages, it is likely that many alleles in the founding population only migrated partially outside of Africa. The results of this study on allelic richness and ancestry variability align with the expectations of models proposing an African origin of humanity, which involve successive founder effects during outward migrations [74]. This finding aligns with a previous study conducted on the X chromosome, which specifically examined the parameter of allelic richness [75].

The Q-matrices derived from Y-STRs have been previously analysed in the genetic landscapes of Africa and the Middle East, encompassing 135 populations and regions, and comprising 11,305 individuals. Within this dataset, 97 African and 38 Middle Eastern populations were investigated, leading to the identification of 8 genetic clusters (K = 8) [70]. Building on this groundwork, the current study broadened its scope to encompass 186 populations (14,504 individuals), including 52 Middle Eastern and 134 African populations leading to the identification of 10 genetic clusters (K = 10). The increased precision and accuracy of the Q-matrix obtained can be attributed to the enhanced demographic and ethnic diversity captured within the expanded survey, emphasising the importance of broader population sampling in elucidating the complex genetic structure of diverse human populations.

The findings of this study revealed that African populations outside Africa (AOA), such as Africans in the UK, USA, Jamaica, the Bahamas, and Haiti, are genetically related to Western and Central African populations. In addition, the phylogenetic analysis showed that these populations merged with the main African cluster. However, the study also highlighted that these populations have formed a distinct genetic cluster that is slightly different from their ancestral groups. This assertion is further substantiated by the comparatively low genetic variability observed within the AOA population, in contrast to other African populations. Additionally, the allelic richness of the AOA population was notably lower than that of other African populations, indicating a deviation from the founder effect commonly observed in African populations. This could be explained by the fact that environmental stresses and social transitions have acted as major selective forces, which may have reshaped the genetic makeup of the African populations inhabiting these regions [76–78].

The aforementioned findings are consistent with the outcomes of a previous study, which revealed that the genetic ancestry of all African Americans is distinguished by admixture in their African elements, primarily originating from West and West-Central Africa. Additionally, the study observed limited variability in these proportions of African heritage among individuals within this demographic [79].

This investigation revealed that the Jewish communities in Libya, Morocco, Yemen, Samaritans, and South Africa exhibit similar genetic structures and ancestral variation to those of Middle Eastern populations. This similarity may be attributed to inter-ethnic marriages among diverse Jewish communities and genetic exchange with the respective host Diaspora populations [80, 81]. Notably, the Ashkenazi Jews stood out as an exception, as clearly demonstrated in the Q matrix and by their significantly low genetic variability. This distinctiveness could be linked to their emergence from migrations northward into the Rhineland from Mediterranean Jewish populations during the early Middle Ages, as well as their prolonged isolation from other communities [82].

This study conducted an analysis of 13 Berber populations located in Egypt, Tunisia, and Morocco. The findings revealed that despite the historical admixture with Arab populations in North Africa, the Berber communities exhibited a distinct and relatively consistent genetic structure, thereby substantiating the hypothesis that Arabs and Berbers possess separate gene pools [83–85]. Notably, an exception was observed in the genetic structure of the Berber population in Egypt, which closely resembled that of the Egyptian Arab population. Additionally, the study of ancestry variability among the Berbers indicated that there was no discernible difference between this population and other North African populations.

This study has demonstrated that the Yemeni population exhibits low ancestry variability in comparison to other Arab populations in the Middle East, suggesting that Yemen may be the origin of Arab populations in the Arabian Peninsula. This finding was highlighted in a previous study [14]; however, our current research, which incorporated more populations, ethnic groups, and regions, yielded ten genetic clusters instead of six and revealed striking differences in genetic ancestry. The use of a higher number of populations and ethnic groups likely allowed for a more detailed examination of genetic variation. This increased granularity captured more subtle differences in genetic ancestry, leading to clearer distinctions between different populations.

The analysis of gene flow has revealed predominant migration patterns within Africa, indicating a consistent trajectory from West Africa to the southern regions, followed by movement from the south to East Africa. Subsequently, gene flow suggests migration from East Africa to Central Africa, and from Central Africa to North Africa. A recent study highlighted the gene flow of Bantu population from West Africa spreading through the Congo rainforest to eastern and southern Africa [86]; however, this study did not trace the migrations of other African populations within the continent.

## Key points

1. The most common haplogroup among Middle Eastern populations was J1a (29.4%), while in African populations, it was E1b1a (43.2%).
2. TMRCA analysis identified Iraq and Yemen as having the most ancient paternal lineages among Middle Eastern populations.
3. Berbers generally form their own distinct genetic cluster, with the notable exception of Berbers in Egypt, who show greater genetic similarity to Egyptian Arabs.
4. Eastern African populations represent the ancestral origin of African populations. They display lower ancestry variability compared to populations in other African regions.
5. Jewish populations generally exhibit genetic structures similar to other Middle Eastern populations, with the significant exception of Ashkenazi Jews. This is supported by both genetic structure analysis and ancestry variability analysis.

## Supplementary Information

Below is the link to the electronic supplementary material.

Supplementary file1 (HTML 3785 KB)

Supplementary file2 (DOCX 1258 KB)

Supplementary file3 (XSLX. 1258 KB)

## Data Availability

All relevant data are within the paper and its Supporting Information files.

## Abbreviations

AMOVA: Analysis of molecular variance
DNA: Deoxyribonucleic acid
FST: Is the proportion of the total genetic variance contained in a subpopulation
GD: Gene diversity
Ia: The amount of information gained about ancestry coefficients from observation of a single randomly chosen allele at a locus.
In: The amount of information gained about population assignment from observation of a single randomly chosen allele at a locus.
MDS: Multidimensional scaling
ORCA: This is the optimal rate of correct assignment for a locus if a randomly chosen allele in a pooled collection of populations is assigned to its most likely source population.
PiXY: The average pairwise differences between individuals from two different populations (X and Y) providing insights into their genetic divergence
PiX: Is the average pairwise difference within population X
PiY: Is the average pairwise difference within population Y
R_ST_: A pairwise population genetic distance measures that incorporate the stepwise mutation process in microsatellites
STR: Short tandem repeat
STRAF: STR analysis for forensics

## Code availability

N/A.

## Declarations

### Clinical Trial number

N/A
