# Supplementary material for: Deciphering the genetic diversity in the Arabian Peninsula and Africa: insights from Y-STR data
Source: Forensic Sci Med Pathol. 2025 Nov 6;22(2):600–18. doi: 10.1007/s12024-025-01115-3 (PMC13331941; doi:10.1007/s12024-025-01115-3)
Supplement: Supplementary file 2 — Supplementary file2 (DOCX 1258 KB) [file 12024_2025_1115_MOESM2_ESM.docx]

| A | 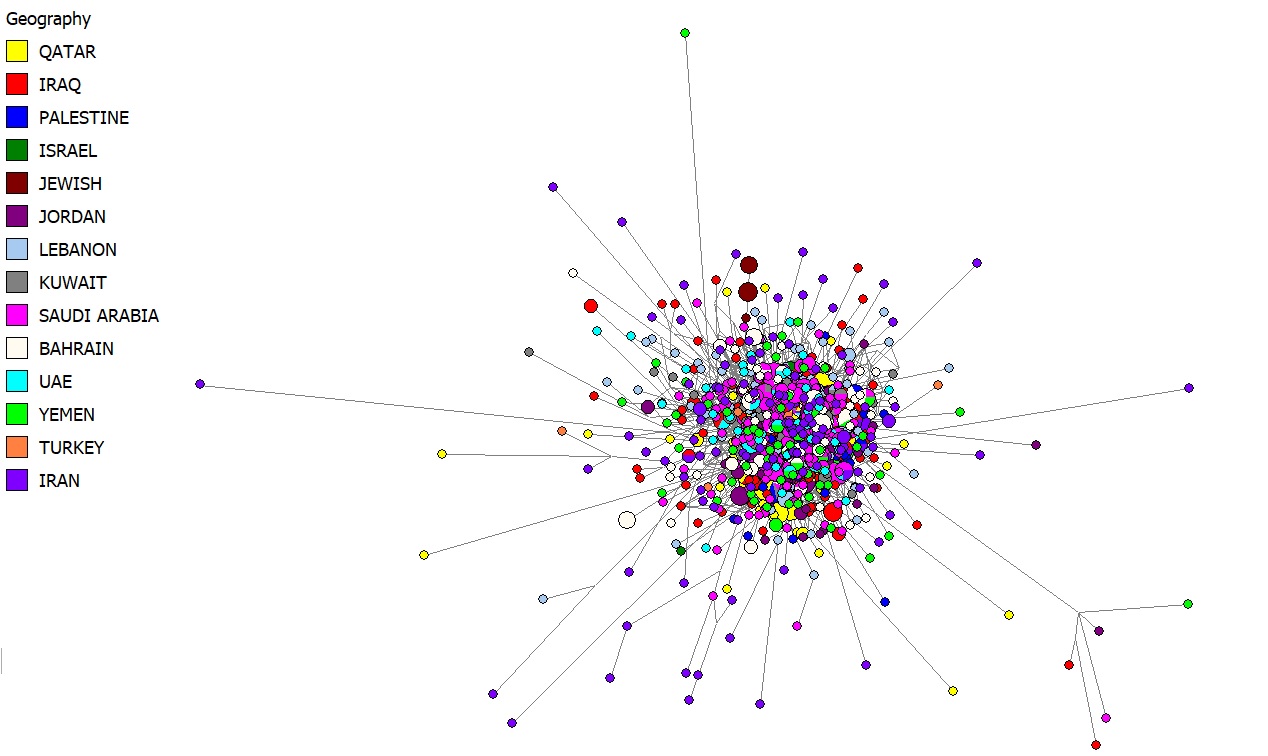 |
| --- | --- |
| B | 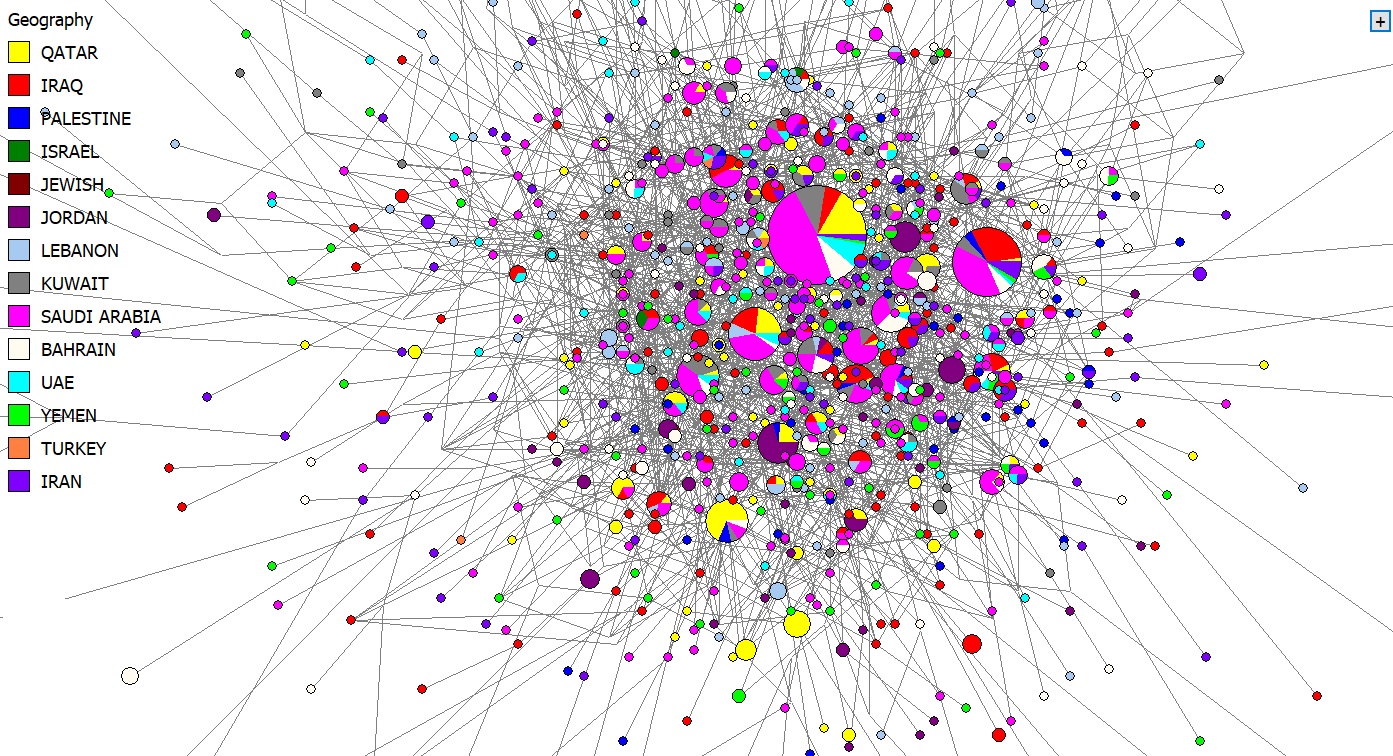 |
| C | 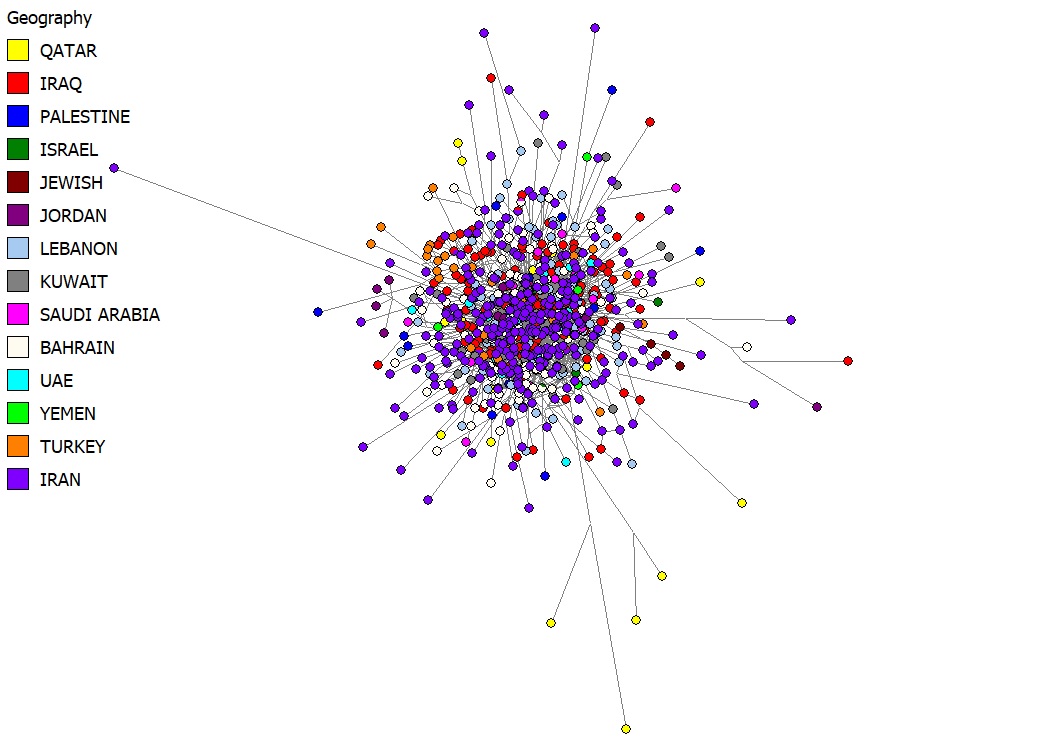 |
| D | 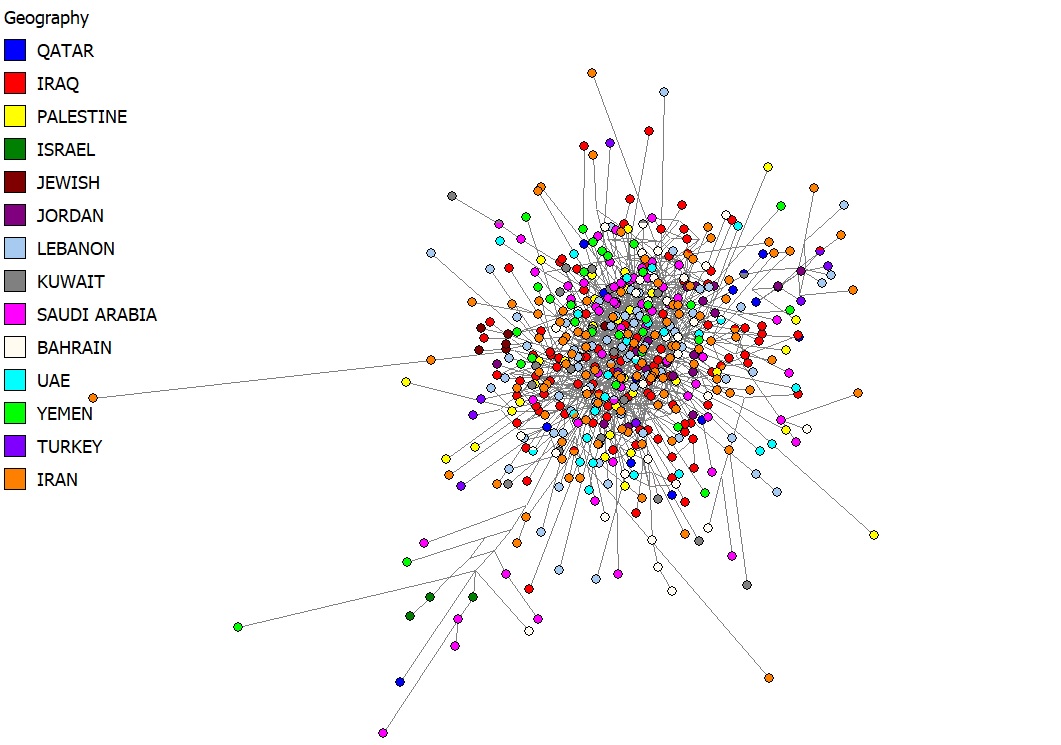 |
| E | 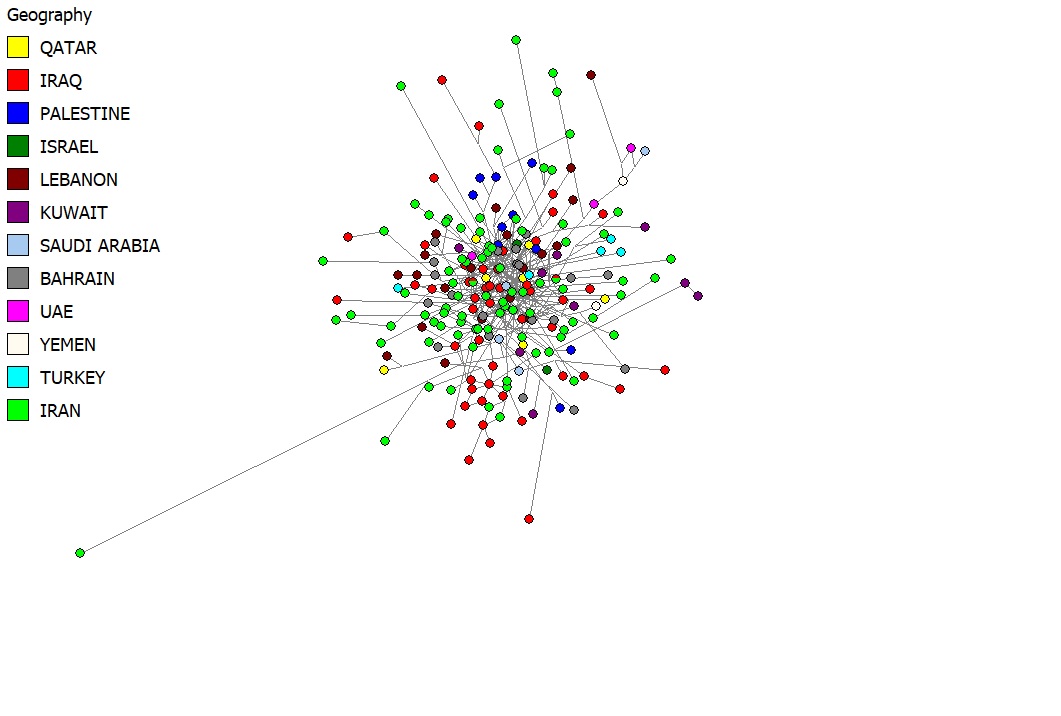 |
| F | 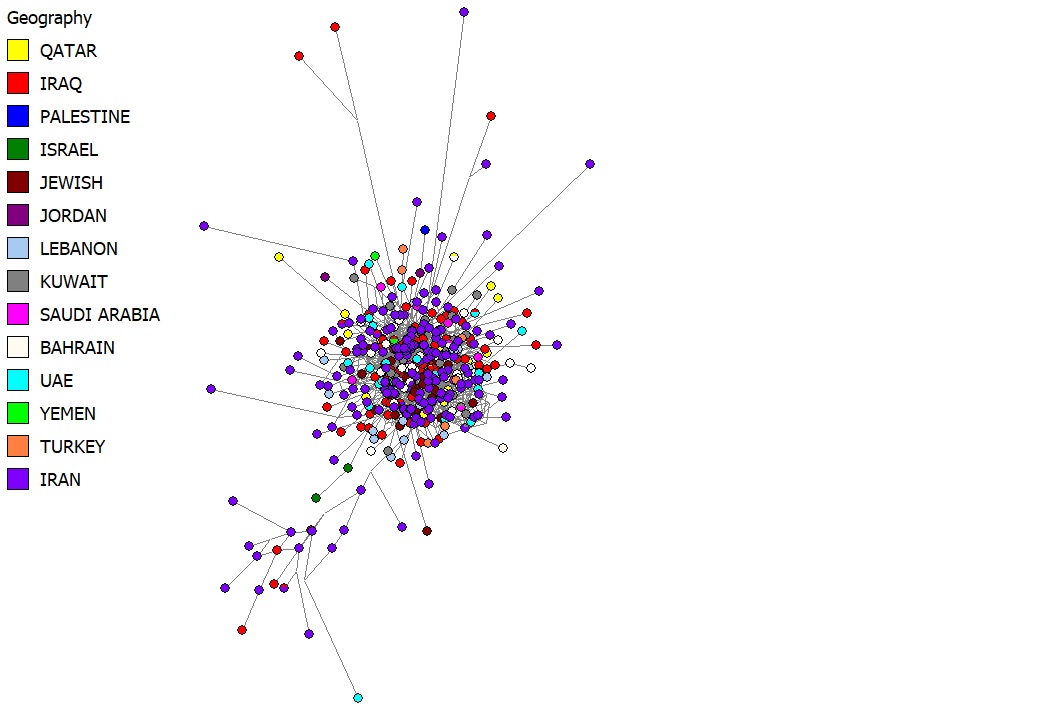 |

**Fig S1 Median-joining networks for the five most common haplogroups in the Middle East.** A and B haplogroup J1a, C. haplogroup J2a, D. haplogroup E1b1b, E. haplogroup G2a and F. haplogroup R1a.
